# Special Issue “Replication and Spread of Alphaherpesviruses”

**DOI:** 10.3390/v14081652

**Published:** 2022-07-28

**Authors:** Stephen A. Rice

**Affiliations:** Department of Microbiology and Immunology, University of Minnesota, Minneapolis, MN 55455, USA; ricex019@umn.edu

Alphaherpesviruses, one of three sub-families of the *Herpesviridae*, are of keen interest to biomedical scientists for several reasons. First, they include several important human and veterinary pathogens. Second, some alphaherpesviruses, such as herpes simplex virus type 1 (HSV-1), replicate robustly in cultured cells and thus make excellent models to study fundamental replication processes. Third, modified alphaherpesviruses are increasingly being developed as novel therapeutics. This Special Issue (SI) of *Viruses* focuses on the replication and spread of alphaherpesviruses in their vertebrate hosts. It consists of 12 papers, 7 of which are primary research articles and 5 of which are reviews. The topics covered fall into four general areas:

1. Replication mechanisms. Three articles deal with alphaherpesvirus replication. Gonzalez del-Pino and Heldwein [1] broadly review herpesviral cell entry, with an emphasis on the role played by a conserved virion glycoprotein complex, the gH/gL heterodimer. gH/gL is key to entry as it regulates the membrane fusogenic activity of a third conserved viral factor, gB. The authors detail how gH/gL functions as a “signal integration machine” that accepts diverse regulatory inputs to trigger herpesviral entry. Another review by Hennig et al. [2] focuses on HSV-1 and how it commandeers the cellular RNA polymerase II machinery for transcription of the viral genome. Included is a discussion of how recent advances in systems biology have led to exciting new findings in this area. Lastly, a review by Packard and Dembowski [3] covers current knowledge of the biochemistry of HSV-1 DNA replication. The authors highlight how HSV-1 adapts and even modifies host factors to assist in viral genome synthesis.
2. Viral spread. Following their replication, alphaherpesviruses must efficiently spread to new target cells and hosts. Three articles in the SI deal with this topic. A review by Rice [4] covers the release of “cell-free” HSV-1, i.e., the delivery of progeny virions directly into the extracellular milieu. The author argues that this type of release is likely critical for human–human transmission and is thus potentially up-regulated in vivo during viral reactivation events in skin and mucosal tissues. Two primary research articles also deal with host–host spread, in this case of avian alphaherpesviruses. The article by Vega-Rodriguez et al. [5] focuses on Marek’s disease virus (MDV) and an MDV glycoprotein, gC, that is required for transmission between chicken hosts. The authors investigate whether gC homologs from related alphaherpesviruses can substitute for MDV gC in mediating spread. The article by Krieter et al. [6] investigates host–host spread in another avian alphaherpesvirus system, gallid alphaherpesvirus 3 (GaHV3), and the potential role of a conserved viral protein kinase.
3. Tropism. During their natural infections, alphaherpesviruses commonly replicate in multiple tissues of their vertebrate hosts. Additionally, these viruses invariably establish latency in particular tissues, such as the peripheral nervous system in the case of HSV-1. However, the determinants of alphaherpesviral tropism are not well understood. The SI contains three papers relating to this important topic. The article by Ostler and Jones [7] focuses on HSV-1 latency in neurons and in particular how the virus can reinitiate productive infection in these cells, often in response to stress. Speculating that the transcription of viral immediate-early (IE) genes is crucial, the authors identify stress-associated cellular transcription factors that can activate a key IE promoter. Another article by Bergstrom et al. [8] looks at the interaction of HSV-1 and its close cousin HSV-2 with the central nervous system (CNS), an important topic since both viruses can cause CNS disease. To study this, the authors differentiate human induced pluripotent stem cells (iPSCs) towards the cortical neuron lineage, examining the replication of HSV-1 in various cell stages along the way. A third paper by Niemeyer et al. [9] focuses on the potential tropism of varicella-zoster virus (VZV), another human alphaherpesviral pathogen, for the adrenal gland. To examine this, the authors study adrenal gland tissue from non-human primates infected with a simian virus closely related to VZV.
4. Therapeutics. Three articles in the SI show how knowledge of alphaherpesviral replication and spread can be harnessed to develop new viral-based therapies. Currently, the most high-profile such therapy is T-Vec, a modified oncolytic HSV-1 strain licensed to treat human cancer. Two articles relate to further development of oncolytic HSV-1. First, Hong et al. [10] provide a timely review of oncolytic HSV-1 strains and how they replicate in, spread through, and destroy tumor tissue. Second, a research article by Kalke et al. [11] points out that T-Vec and other HSV-1-based therapeutics have been developed using only a limited set of parental strains. To help to remedy this potential limitation, the authors characterize a large set of HSV-1 clinical strains with the aim of identifying ones which have improved spread and tumor-cell-killing properties. A third article by Bhattacharya et al. [12] investigates a completely novel alphaherpesviral-based therapeutic strategy. The authors purify plasma-membrane-derived liposomes from HSV-1-infected cells and show that these entities have a surprising degree of anti-HSV-1 activity, perhaps by inhibiting viral entry.

As Guest Editor, I am extremely pleased with the strong collection of articles in “Alphaherpesvirus Replication and Spread”, and I invite you to delve deeply into the impressive science within. I believe that the collected articles well illustrate the excitement and vigor that permeate current research into the fundamental biology of these important viruses. Additionally, this collection reveals how the study of alphaherpesviral replication and spread can be translated into important new advances in human and veterinary medicine.
